# Programming adaptive laboratory evolution of 4-hydroxyisoleucine production driven by a lysine biosensor in *Corynebacterium glutamicum*

**DOI:** 10.1186/s13568-021-01227-3

**Published:** 2021-05-08

**Authors:** Xinping Yu, Feng Shi, Haiyan Liu, Shuyu Tan, Yongfu Li

**Affiliations:** 1 State Key Laboratory of Food Science and Technology, Jiangnan University, 1800 Lihu Avenue, Wuxi, 214122 China; 2 Key Laboratory of Industrial Biotechnology, Ministry of Education, School of Biotechnology, Jiangnan University, Wuxi, 214122 China; 3 International Joint Laboratory On Food Safety, Jiangnan University, Wuxi, 214122 China; 4 National Engineering Laboratory for Cereal Fermentation Technology, Jiangnan University, Wuxi, 214122 China

**Keywords:** 4-Hydroxyisoleucine, *Corynebacterium glutamicum*, Adaptive laboratory evolution, Programming evolution, LysG-P_*lysE*_, Lys biosensor

## Abstract

4-Hydroxyisoleucine (4-HIL) is a promising drug for treating diabetes. In our previous study, 4-HIL was synthesized from self-produced L-isoleucine (Ile) in *Corynebacterium glutamicum* by expressing an Ile dioxygenase gene. Although the 4-HIL production of recombinant strain SZ06 increased significantly, a by-product, L-lysine (Lys) was accumulated because of the share of the first several enzymes in Ile and Lys biosynthetic pathways. In this study, programming adaptive laboratory evolution (ALE) was designed and conducted in SZ06 to promote 4-HIL biosynthesis. At first, a programming evolutionary system pMK was constructed, which contains a Lys biosensor LysG-P_*lysE*_ and an evolutionary actuator composed of a mutagenesis gene and a fluorescent protein gene. The evolutionary strain SZ06/pMK was then let to be evolved programmatically and spontaneously by sensing Lys concentration. After successive rounds of evolution, nine mutant strains K1 − K9 with significantly increased 4-HIL production and growth performance were obtained. The maximum 4-HIL titer was 152.19 ± 14.60 mM, 28.4% higher than that in SZ06. This titer was higher than those of all the metabolic engineered *C. glutamicum* strains ever constructed. The whole genome sequencing of the nine evolved strains revealed approximately 30 genetic mutations in each strain. Only one mutation was directly related to the Lys biosynthetic pathway. Therefore, programming ALE driven by Lys biosensor can be used as an effective strategy to increase 4-HIL production in *C. glutamicum*.

## Key points

- Lys-sensing evolution system was designed to drive programming ALE in *C. glutamicum*.
- Programming evolution based on Lys biosensor was applied to improve 4-HIL production.
- Positive mutants with higher 4-HIL titer were quickly obtained via programming ALE.

## Introduction

According to the ninth edition of the Diabetes Atlas published by the International Diabetes Federation in 2019, more than 463 million adults are suffered from diabetes worldwide, and this number will reach 578.4 million by 2030 and up to 700.2 million by 2045 (Saeedi et al. 2019). Among these populations, the populations with type 2 diabetes mellitus (T2DM) accounts for approximately 90%. These patients are characterized by various degrees of insulin deficiency and extensive insulin resistance. Encouragingly, (2*S*, 3*R*, 4*S*)-4-hydroxyisoleucine (4-HIL), which was found in fenugreek (*Trigonella foenum*-*graecum*) (Neelakantan et al. 2014), exhibits the particular glucose-dependent insulin-stimulating and insulin-sensitizing activities. Thus, 4-HIL becomes a promising drug for treating the T2DM (Zafar and Gao 2016). Traditionally, 4-HIL is extracted from fenugreek seeds, but its yield is only 150 mg/kg (Jetté et al. 2009). Fortunately, the α-ketoglutarate (α-KG)-dependent L-isoleucine dioxygenase (IDO) derived from *Bacillus thuringiensis* was found to specifically convert L-isoleucine (Ile) to 4-HIL (Ogawa et al. 2011). 4-HIL was then synthesized by overexpressing the IDO gene (*ido*) in *Escherichia coli*, however, the addition of Ile makes such process costly and inefficient (Smirnov et al. 2010).

*Corynebacterium glutamicum* is globally recognized as safe and is widely used in the production of various amino acids (Becker et al. 2018; Tsuge and Matsuzawa 2021). In our previous study, through overexpressing *ido* gene derived from *B. thuringiensis* YBT-1520 in an Ile-producing *C. glutamicum* ssp. *lactofermentum* strain SN01, 65.44 ± 2.27 mM 4-HIL was synthesized from the self-produced Ile (Shi et al. 2015). In order to improve 4-HIL production, metabolic engineering was performed, such as synergistically enhancing the substrates supply and IDO activity by particular gene overexpression and deletion (Shi et al. 2016, 2018, 2019). The engineered strain SZ06 could produce 111.99 ± 2.15 mM 4-HIL (Shi et al. 2019). Although the 4-HIL production of SZ06 increased significantly, a by-product, L-lysine (Lys) was also accumulated, because the first several enzymes of the Ile biosynthetic pathway are involved in Lys biosynthesis simultaneously. Furthermore, diaminopimelate, an intermediate metabolite of the Lys biosynthetic pathway, is required for cell growth, making it difficult to weaken the Lys biosynthesis. The accumulation of Lys limits the further increase of 4-HIL production.

Adaptive laboratory evolution (ALE) has become a powerful strategy in metabolic engineering. Compared with traditional metabolic design, ALE generates non-intuitive beneficial mutations that can occur in many different gene regions simultaneously under specific selection pressures (Portnoy et al. 2011). This natural selection provides an effective basis for obtaining new characteristics, phenotypes, and beneficial mutations in microorganisms, which can be fixed after multiple rounds of continuous evolution (Desai and Fisher 2007). It is precisely because of the rapid adaptation of microorganisms to different environments, the application of ALE for phenotypic optimization has been extended to various aspects, such as isolating the best strains, activating latent pathways, and improving environmental tolerance of production strains, etc. (Portnoy et al. 2011; Sandberg et al. 2019). This phenotypic optimization not only improves overall cell function but also improves the physiological adaptation of microbial strain. Because normally, the adaptive rate under specific selection pressures is directly coupled to the growth rate, this coupled metabolic design will result in significantly higher production and lower by-products (Fong et al. 2005; Barrick et al. 2009). For example, ALE was successfully applied to improve cellular tolerance of a *C. glutamicum* strain to high concentrations of methanol, thereby enhancing methanol biotransformation, meanwhile, the cell growth was also improved (Tuyishime et al. 2018; Wang et al. 2020). ALE was used to develop potential evolutionary strains of *Zymomonas mobilis* that could co-utilize glucose and xylose (Millán et al. 2020). Besides lowering effects by toxic chemicals, ALE has also been successfully used to improve production with *C. glutamicum*. For example, ALE combined with rare codon-rich markers was applied to select a mutant strain with 3.7-fold increased production of L-arginine (Arg) (Zheng et al. 2018). ALE combined with metabolic engineering was successfully used to improve the production of putrescine in *C. glutamicum* (Li et al. 2018). Through ALE driven by an L-valine (Val) biosensor Lrp-P_*brnFE*_ in combination with atmospheric and room temperature plasma mutagenesis, an mutant strain HL2-7 was successfully obtained and its Val production increased by 21.47% (Han et al. 2020).

However, when the selection pressure coupled to growth is not available, the desired mutant strains are difficult to be screened out by ALE. In addition, the mutation rate is too low when ALE is performed under natural evolution. Considering these two problems, ALE was ameliorated by using feed-back regulated evolution of phenotype (FREP) to increase the mutation rate and the population diversity (Binder et al. 2013). The FREP is actually a programming adaptive control system, in which the mutation rate is improved under the initial condition and decreased under the final condition when the concentration of target metabolites increased, thus resulting in higher positive screening rate (Chou and Keasling 2013). FREP is implemented in two parts: a biosensor for sensing the concentration of the target metabolite to generate the transcriptional signal and an actuator for changing the mutation rate when receives transcriptional signal. In this case, the sensor consists of two components: a transcription factor (TF) that binds to the target metabolite and a promoter that is regulated by the TF. The actuator is usually a mutagenesis gene, which can shorten the evolution time. The effect of FREP was successfully verified by evolving *E. coli* strains to increase the production of tyrosine and isoprenoid, meanwhile, a fluorescent protein, another actuator, was expressed in order to detect the visible changes in the evolved strains (Chou and Keasling 2013). Delightfully, more and more researches have been conducted in the field of TF-based biosensors. However, sensors that can be used for metabolic engineering of *C. glutamicum* is limited, for example, the branched-chain amino acids (BACCs) and L-methionine biosensor Lrp-P_*brnFE*_ (Mustafi et al. 2012; Tan et al. 2020), Lys, L-histidine (His), and Arg biosensor LysG-P_*lysE*_ (Schendzielorz et al. 2014; Kortmann et al. 2019), and shikimic acid biosensor ShiR-P_*shiA*_ (Liu et al. 2017).

*lysE* encodes the Lys, His, and Arg export protein, while LysG, an LysR-Type TF, is a positive regulator of *lysE* transcription. In the presence of intracellular Lys, Lys binds to LysG, leading LysG to bind in the promoter region of *lysE*, thereby promoting the transcription of *lysE* (Bellmann et al. 2001). Recently, an optical Lys sensor pSenLys was constructed based on LysG-P_*lysE*_, and several new Lys producer were generated by fluorescence-activated cell sorting (FACS) of mutant strains harboring this sensor (Binder et al. 2013). Subsequently, semi-conserved LysG was designed to construct the biosensor pSenHis with focused His and Arg specificity and the His-producing mutant strains of *C. glutamicum* was obtained through this pSenHis-based FACS (Della Corte et al. 2020).

In this study, in order to improve the 4-HIL production and reduce the by-product Lys production, a programming ALE driven by the Lys-sensing evolution system was employed. Firstly, a Lys-sensing evolution system pMK was constructed. It containes two parts: the Lys biosensor LysG-P_*lysE*_ and the actuator composed of a mutagenesis gene *cdd* and a fluorescent reporter gene *egfp*. *cdd* gene encodes the cytosine deaminase. It has been used in genome editing to cause specific mutations at target sites and generate gene silencing (Banno et al. 2018; Wang et al. 2019). *cdd* was used here to accelerate mutation and evolution. Thereby, the mutation rate and eGFP fluorescence intensity of cells may be positively regulated in this system according to the intracellular Lys concentration. Secondly, the evolutionary strain SZ06/pMK was constructed and programming ALE was conducted to generate the evolved positive mutants. Finally, the whole genome of these mutant strains was sequenced and the mutations in these evolved strains were analyzed.

## Materials and methods

### Strains, plasmids, and culture conditions

The strains and plasmids used in this study are listed in Table 1. *E. coil* JM109 was used for gene cloning and propagation. *E. coil* cells were grown in Luria–Bertani (LB) medium at 37 °C and 200 rpm. *C. glutamicum* ssp. *lactofermentum* SZ06 was used as the initial strain for 4-HIL production. *C. glutamicum* was cultivated in LBB medium (5 g/L trypone, 2.5 g/L yeast extract, 5 g/L NaCl, and 18.5 g/L brain heart infusion powder) at 30 °C and 200 rpm. If necessary, 30 μg/mL kanamycin and 10 μg/mL chloramphenicol was added to the media to promote plasmid maintenance.

**Table 1:** Bacterial strains and plasmids used in this study

Strains or plasmids	Description	Source
Strains		
JM109	Plasmid propagating strain of *E. coli*	Novagen
SZ06	Ile-producing strain of *C. glutamicum* ssp. *lactofermentum*	Shi et al. 2019
SZ06/pMK	SZ06 harboring pMK	This work
MKn	Positively evolved mutant strains of SZ06/pMK	This work
Kn	Final evolved strains of MKn that lose pMK	This work
Plasmids		
pJYW-4	Constitutive expression vector of *C. glutamicum*, Km^R^	Hu et al. 2014
pDTW109	*cre*-expressing vector of *C. glutamicum*, temperature-sensitive, Cm^R^	Hu et al. 2013
pMK	pDTW109 harboring *lysG* and P_*lysE*_ controlled *cdd*-*egfp* genes, Cm^R^	This work

### Construction of evolution plasmid and *C. glutamicum* strain for ALE

A Lys-sensing evolution plasmid containing Lys biosensor *lysG*-P_*lysE*_ and evolution actuator *cdd*-*egfp* was constructed as follow. The primers used in this study are listed in Table 2. The *lysG*-P_*lysE*_ fragment and *cdd* gene were amplified from the genome of strain SN01. Then they were fused with chemically synthesized *egfp* gene by overlap PCR. Next, the PCR product *lysG*-P_*lysE*_-*cdd*-*egfp* was digested with *Sal*I and *Pst*I and ligated into the *Sal*I- and *Pst*I-digested plasmid pDTW109, generating the Lys-driven evolution plasmid pMK. Finally, the pMK was transformed into *C. glutamicum* SZ06, generating the evolutionary strain SZ06/pMK.

**Table 2:** Primers used in this study

Primers	Sequence (5’-3’)	Restriction sites
*lysG*-P_*lysE*_-R	TAAT**GTCGAC**TTAAGGCCGCAATCCCTCG	*Sal*I
*lysG*-P_*lysE*_-F	ACCATCCTATAACTCCTTCTCGGTCCGATGGACAGCAAAAG	
*egfp*-F	AGAAGGAGTTATAGGATGGTTTCCAAGGGCG	
*egfp*-R	TTACTTGTACAGCTCGTCC	
*cdd*-F	AGTAAGAGAGGAGGGATTGCATGACTGAAGATGACTTAGATCTG	
*cdd*-R	AGTA**CTGCAG**CTAAAGAGCCTTATCCGGAG	*Pst*I
16 s-RNA-1	ACCTGGAGAAGAAGCACCG	
16 s-RNA-2	TCAAGTTATGCCCGTATCG	
*dapC*-1	TCTTATGTGGGGGCTACACC	
*dapC*-2	CATCACGATCCCCAATATCC	
CGB98_RS08395-1	GGTCCTAACGCTTTCGACTG	
CGB98_RS08395-2	GCTGTTCAGTGCGTGGATAA	
CGB98_RS10415-1	TGTCAATAACTCGCCTGGTG	
CGB98_RS10415-2	CAGTGCATCAACGTGGACTT	
*actA*-1	TGCAACCTCCTTCTCCTTGT	
*actA*-2	TGCCATAGATGTCAGCTTCG	
CGB98_RS10920-1	TGCACGTAAAGACGGAGTTG	
CGB98_RS10920-2	GTTGCCGTAGATGTCGTTGA	

Restriction sites are indicated in boldface

### Adaptive laboratory evolution

**Fig. 1:**
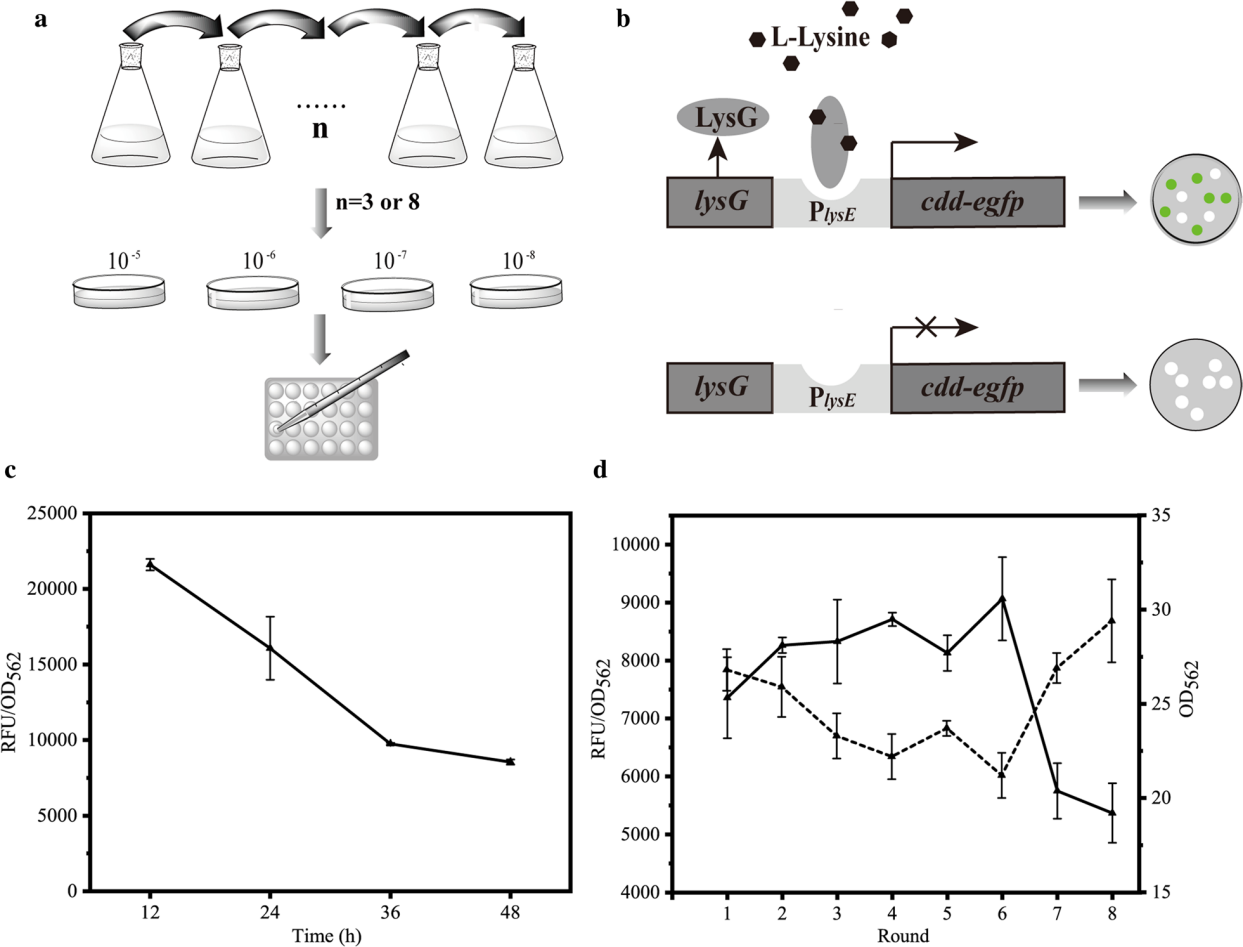
The Lys-biosensor based programming ALE process. The Lys biosensor senses the intracellular Lys concentration and regulates the expression level of the evolution actuator, thereby controls and indicates the evolution process. **a** Schematic of programming ALE process. The evolutionary strain SZ06/pMK was repeatedly and continuously cultured for several rounds in the same medium, the evolution process was ceased and the evolved cells were harvested when the relative eGFP fluorescence intensity no longer reduced significantly. Then, 22 single colonies were picked randomly for purification, and they were cultured in 24 deep-well plate. **b** The work diagram of pMK. When Lys concentration is higher, the biosensor was activated, thereby activating the expression of *cdd* and *egfp* and increasing the mutation rate and the eGFP fluorescence intensity of cells. When Lys concentration is lower, the Lys biosensor will not be activated, the relative eGFP fluorescence intensity will be decreased and the evolution process was ceased. **c** The change of relative eGFP fluorescence intensity in the 3^rd^ round. **d** The relative eGFP fluorescence intensity (solid line) and biomass (dotted line) of SZ06/pMK at 48 h of each round

The evolutionary strain SZ06/pMK was precultured in the seed medium for 18 h, inoculated into fermentation medium as described previously (Shi et al. 2019), and then cultured and evolved spontaneously for 48 h at 30 °C, 200 rpm. Subsequently, 4% volume of cultured broth was transferred into fresh fermentation medium to proceed a new round of evolution under the same conditions (Fig. 1a). During evolution, 1 mL broth was harvested every 12 h to detect eGFP fluorescence intensity. The evolution process was ceased and the evolved cells were harvested when the relative fluorescence intensity no longer reduced. After dilution, the cells were spread onto LBB plates and incubated at 30 °C for 36 h, then 22 single colonies were picked randomly for purification. Next, they were pre-cultured and then cultured in 24 deep-well plate with 2 mL medium in each well. The amino acid concentration was detected as described below to select the positively evolved strains MKn. Finally, the MKn were cultivated at 37 °C for 24 h to cure pMK and to obtain pMK-free strains Kn.

### Fluorescence assays

The fermentation broth of SZ06/pMK was diluted by 1 M hydrochloric acid and then transferred into a black 96-deep-well plate with clear bottom (Corning-Costar plates) for detection. Before each test, the plate was vibrated in Cytation5 (BiotTek) for 30 ms. The biomass was detected at 562 nm and the eGFP fluorescence was determined at an excitation wavelength of 479/20 nm and an emission wavelength of 520/20 nm. When characterizing the sensor, Lys was added into the well to a final concentration of 0, 5, 10, 20, and 30 mM, and fluorescence was measured as above after incubating the plate in the Cytation5 for certain time.

### 4-HIL fermentation

For 4-HIL fermentation in a shake flask, cells were precultured, inoculated into optimized fermentation medium and then cultured as described previously (Shi et al. 2019). One mL of fermentation broth was harvested every 24 h to measure the cell density, pH, residual glucose, and amino acids and 4-HIL concentrations by the methods described previously (Shi et al. 2018). The conversion ratio of Ile to 4-HIL was calculated as the mole of 4-HIL divided by the total moles of Ile and 4-HIL. The amino acids and 4-HIL concentrations were detected by HPLC analysis by the method described previously (Shi et al. 2016).

### Genome sequencing and analysis of genetic mutations

The genome of mutant strains was sequenced using an Illumina HiSeq 4000 system (San Diego, CA, USA) at the Beijing Genomics Institute (Shenzhen, China) and compared with the reference genome of *C. glutamicum* WM001 (NCBI: GCF_002220135.1). Genomic DNA was sheared randomly to construct nine read libraries with lengths of 240 bp by a Bioruptor ultrasonicator (Diagenode, Denville, NJ, USA) and physicochemical methods. The paired-end fragment libraries were sequenced according to the Illumina HiSeq 4000 system’s protocol after the low abundant sequences were filtered. Raw reads of low quality from paired-end sequencing were discarded.These quenched reads were assembled using SOAP de novo v1.05 software. Each query sequence was then aligned with the reference sequence using the alignment softwares MUMmer (http://mummer.sourceforge.net/) and LASTZ (http://www.bx.psu.edu/miller_lab/dist/README.lastz-1.02.00/) to get the alignment results (Kurtz et al. 2004; Harris 2007). The variation sites between the query sequence and reference sequence were found out and filtered preliminarily to detect single nucleotide polymorphisms (SNP) and insertion-deletion (InDel) sites.

### RT-PCR analysis of genes’ transcription

Real-time PCR (RT-PCR) was used to analyze transcription levels of target genes of mutant strains during fermentation. The samples collected at the mid-exponential phase were used for RT-PCR experiments and the methods were performed as described previously (Chen et al. 2015; Wang et al. 2015). The relative abundance of mRNA of target genes was quantified by measuring the cycle threshold (Ct) value and calculating the 2^−ΔΔCt^ value. Primers for RT-PCR are summarized in Table 2.

## Results

### Characterization of Lys biosensor LysG-P_***lysE***_

**Fig. 2:**
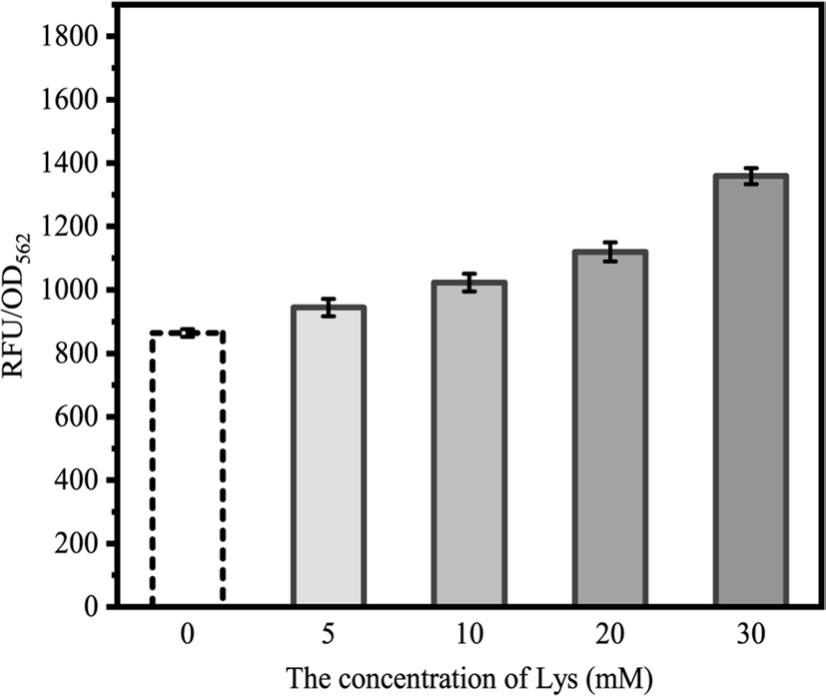
The response of the native LysG-P_*lysE*_ biosensor in pMK to Lys. The relative eGFP fluorescence intensity of evolutionary strain SZ06/pMK was detected under different concentrations of Lys

The natural LysG-P_*lysE*_ biosensor was applied in several studies to monitor the cellular production of Lys, Arg, and His and to screen their better producer (Binder et al. 2012; Schendzielorz et al. 2014; Eggeling and Bott 2015). In our designed programming evolution system here, the natural LysG-P_*lysE*_ was used as the Lys biosensor. When Lys concentration is high, the Lys biosensor will be activated, thereby activating the expression of *cdd* and *egfp* and increasing the mutation rate and the eGFP fluorescence intensity of cells (Fig. 1b). When Lys concentration is lower, the Lys biosensor will not be activated, thereby weakening the expression of *cdd* and *egfp* and decreasing the mutation rate and the relative eGFP fluorescence intensity. Before performing the programming ALE, the sensitivity and response range of the natural LysG-P_*lysE*_ biosensor in this SZ06/pMK strain was characterized. Lys was added into the cell suspension of SZ06/pMK until 5, 10, 20, and 30 mM. The relative eGFP fluorescence intensity increased significantly as the Lys concentration increased from 5 to 30 mM (Fig. 2), suggesting the positive correlation between the expression level of LysG-P_*lysE*_ biosensor-controlled *egfp* and the Lys concentration in the range of 5 − 30 mM. A previous research also verified that 30 − 40 mM Lys was sufficient to induce the expression of *lysE* (Bellmann et al. 2001). The response of the biosensor to Lys ranged from 1.09- to 1.37-fold. Therefore, this evolution system can respond to the varied Lys concentration and can then be applied for programming ALE.

### Programming adaptive laboratory evolution of SZ06/pMK

**Fig. 3:**
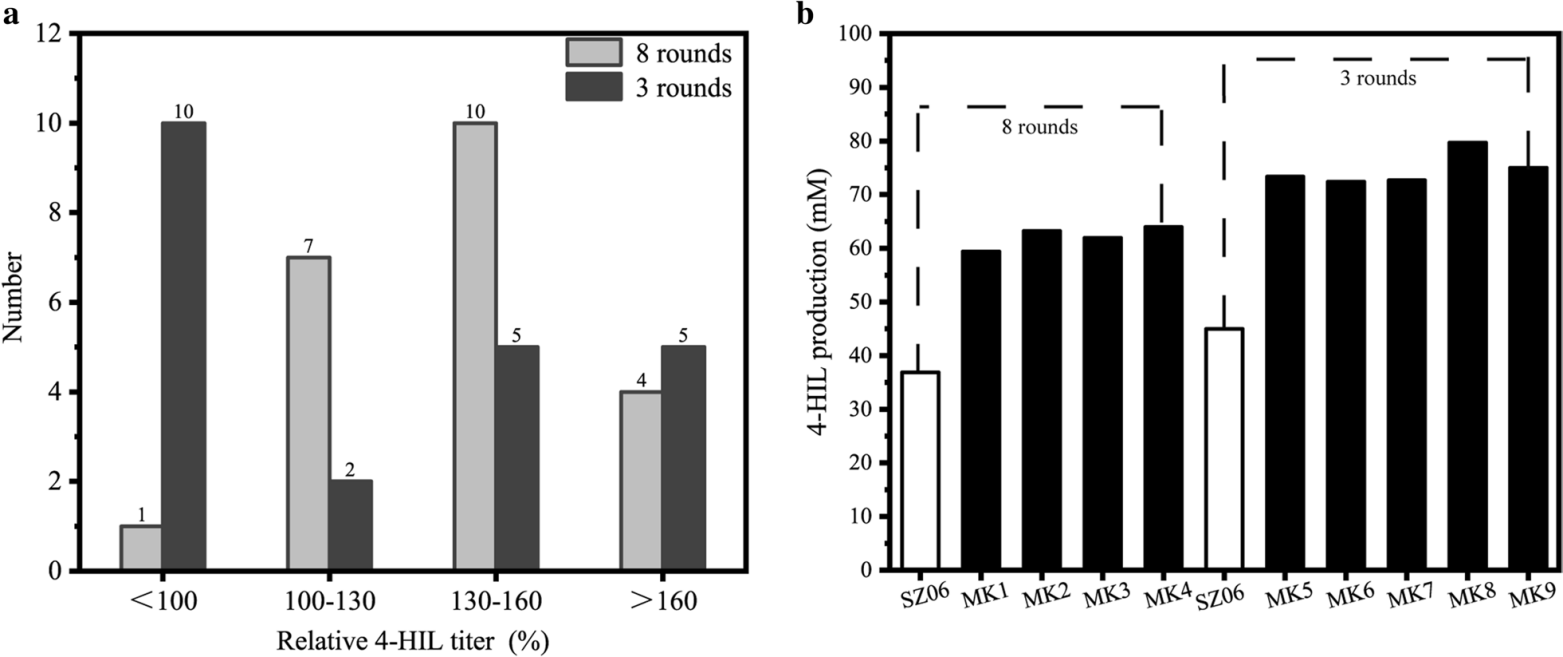
The 4-HIL production of preliminary screening strains in 24 deep-well plates after fermented for 96 h. SZ06 was used as the control strain. **a** The distribution of relative 4-HIL production of mutant strains obtained by eight rounds or three rounds’ evolution. **b** mutant strains with a relative titer higher than 160%, MK1 − MK4 from the eighth round and MK5 − MK9 from the third round, respectively

The strain SZ06/pMK evolved continuously for several rounds (Fig. 1a). At each round, the relative eGFP fluorescence intensity was measured to judge the destination of evolution. The relative eGFP fluorescence intensity decreased continuously during evolving within each round (Fig. 1c), whereas the final fluorescence of each round increased slightly in the first several rounds of evolution and dropped significantly at the seventh round (Fig. 1d). Meanwhile, the final biomass of each round decreased slightly in the first several rounds and increased at the seventh and eighth rounds. After eight rounds, the final fluorescence intensity dropped to 5370 and nearly did not decrease. Thereby, the evolution was ceased and the evolved individual strains were separated on plate. After separation, 22 single colonies were randomly selected and the 4-HIL production of these mutants was detected. 21 mutants showed significantly higher 4-HIL titer (43.31 − 63.98 mM) than the initial strain SZ06 (36.90 mM), thereby the positive mutation rate reached 95.5% (Fig. 3a). The 4-HIL titer of 4 mutant strains, i.e. MK1, MK2, MK3, and MK4 increased to more than 160.0% of that of SZ06 (Fig. 3b). However, the Lys titer (11.44 − 15.77 mM) did not reduce significantly as compared to SZ06 (9.29 mM).

Some studies have utilized ALE to force strains to adapt to alternative growth environments, thus accelerating the growth rate of cells and obtaining mutants with significantly improved performance (Lee and Palsson 2010; Tuyishime et al. 2018; Wang et al. 2020). The shortest evolution period of most ALE was 15 days (Stella et al. 2019), comparable with 8 rounds evolution here. Considering the accelerated and programming evolution in our pMK system, we attempted to shorten the evolution time of programming ALE and to test if we can obtain desired positive mutant strains more quickly.

The programming evolution of strain SZ06/pMK was performed again as previously for only three rounds. 22 mutant strains were randomly screened and their 4-HIL production was determined. 12 mutant strains produced more 4-HIL (54.93 − 79.67 mM) than SZ06 (44.99 mM), and the positive mutation rate reached 54.5% (Fig. 3a). The 4-HIL titer of 5 mutants, i.e. MK5, MK6, MK7, MK8, and MK9, was 72.41 − 79.67 mM, more than 160.0% of that of SZ06 (Fig. 3b). The Lys titer (6.75 − 16.52 mM) did not decrease obviously as compared to SZ06 (5.98 mM). These results demonstrate that the Lys-sensing evolution system can control the programming evolution by sensing Lys concentration. Meanwhile, positive mutants with higher 4-HIL production can be obtained in much shorter evolution time.

### 4-HIL production of envolved mutants

**Fig. 4:**
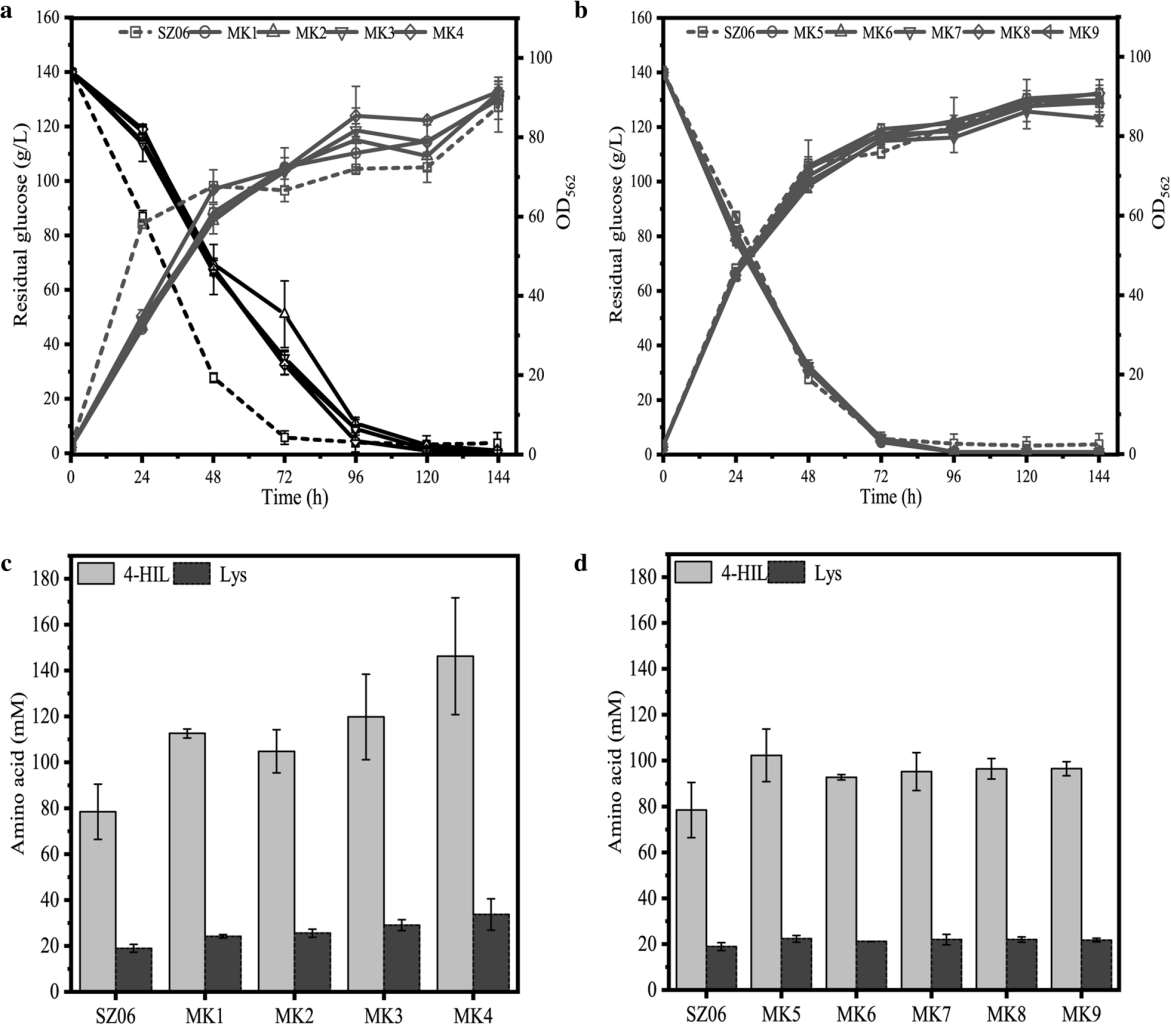
The 4-HIL fermentation of mutant strains MK1 − MK9 in shake flasks for 144 h. SZ06 was used as a control strain. **a** Cell growth and glucose consumption of MK1 − MK4, **b** Cell growth and glucose consumption of MK5 − MK9, **c** 4-HIL and Lys concentration of MK1 − MK4, **d** 4-HIL and Lys concentration of MK5 − MK9

The 4-HIL production of the nine mutants MK1 − MK9 was then evaluated in shake flask. In consistent with the result obtained in 24 deep-well plate, all the nine mutant strains showed higher 4-HIL titer than the control strain SZ06; meanwhile, they exhibited similar growth curves and glucose consumption curve (Fig. 4). The final biomass and glucose consumption of MK1 − MK4 were a little higher than that of SZ06 (Fig. 4a), while those of MK5 − MK9 were nearly same as SZ06 (Fig. 4b).

After 144 h fermentation, 4-HIL production of MK1 − MK4 increased to 104.82 − 146.29 mM (Fig. 4c), while that of MK5 − MK9 increased to 92.72 − 102.31 mM (Fig. 4d). Although 1.33 − 18.85 mM Ile was remained in MK1 − MK9 after fermented in 24-well plates, nearly no Ile was remained after fermented in shake flasks and the conversion ratio of Ile to 4-HIL was almost close to 100.0%. Among these 9 mutants, the maximum 4-HIL titer was 146.30 ± 25.47 mM, which was 89.5% higher than the control strain SZ06 (78.44 ± 12.08 mM). However, the concentration of by-product Lys of these 9 mutants (21.14 − 33.70 mM) increased as compared to SZ06 (18.93 ± 1.74 mM) (Fig. 4c and d).

**Fig. 5:**
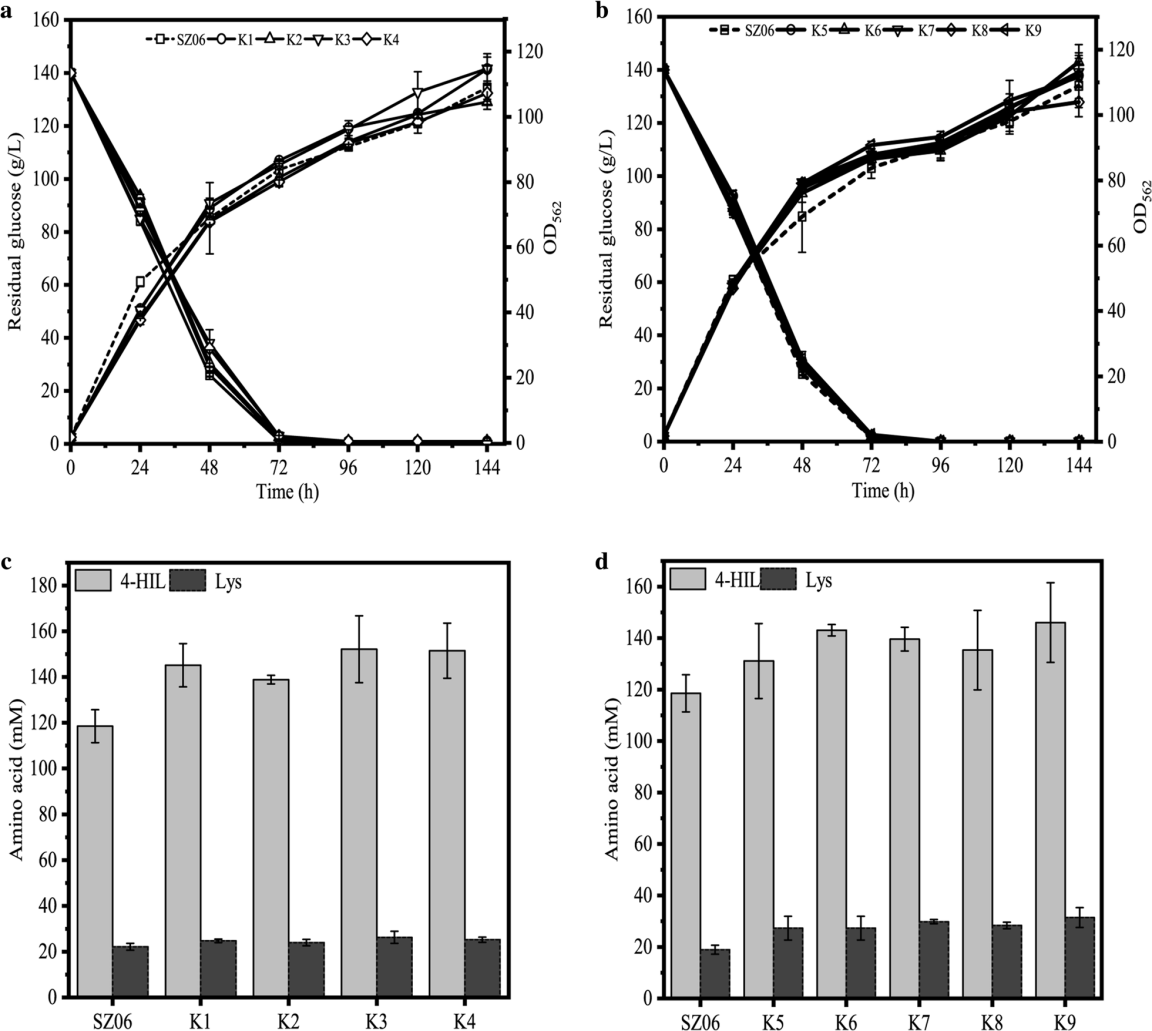
The 4-HIL fermentation of mutant strains K1 − K9 in shake flasks for 144 h. SZ06 was used as a control strain. **a** Cell growth and glucose consumption of K1 − K4, **b** Cell growth and glucose consumption of K5 − K9, **c** 4-HIL and Lys concentration of K1 − K4, **d** 4-HIL and Lys concentration of K5 − K9

To obtain the genetically stable strains after programming ALE, the pMK plasmid was cured in MK1 − MK9. The final evolved strains K1 − K9 were then fermented in shake flasks. The growth and glucose consumption curves of all the final evolved strains were similar to those of SZ06 (Fig. 5a and b). Meanwhile, the final biomass of K1, K3, and K6 was nearly 5.0% higher than that of SZ06, and all glucose was exhausted at 72 h by K1 − K9.

Furthermore, K1 − K9 accumulated 128.87–152.19 mM 4-HIL, which was 10.6–28.4% higher than SZ06 (118.56 ± 7.21 mM) (Fig. 5c and d). Particularly, K3 produced most 4-HIL (152.19 ± 14.60 mM) among the eight rounds-evolved K1 − K4 strains, while among the three rounds-evolved K5 − K9 strains, K9 produced most 4-HIL (146.05 ± 15.48 mM), 28.4% and 23.2% higher than that of SZ06, respectively. Similarly, the concentration of by-product Lys of K1 − K9 (23.98 − 31.42 mM) increased slightly. Recently, the optimal dynamic control strain ST17 and RBS fine-tuning strain SF12 accumulated 135.34 ± 12.55 mM and 139.82 ± 1.56 mM 4-HIL, respectively in shake flask (Shi et al. 2020; Tan et al. 2020). SF12 was nearly the highest titer so far. Pleasurably, the 4-HIL production of K3 was 8.1% higher than that of SF12. The result demonstrates that the programming ALE driven by Lys biosensor can successfully generate *C. glutamicum* mutant strains with higher 4-HIL production.

### Genetic mutations of the evolved strains

Finally, the whole-genome sequencing of K1 − K9 strains was conducted in order to identify mutations in their genome. An average of 32 mutations were revealed in each strain. The common mutation sites of these 9 strains contained 3 SNPs and 6 InDel, among them, 4 mutations were located in the coding regions, i.e. CGB98_RS02465, CGB98_RS09630, and CGB98_RS12800 encoding a 16S ribosomal RNA, a HNH endonuclease (endonuclease Dnase I/II), and IS6 family transposase, respectively (Table 3). The 16S ribosomal RNA is involved in the protein translation. It also affects the stem-loop structure of mRNA, thus affecting gene expression and growth performance of cells. The IS6 family transposase allows genes to change position. However, the influence of these mutation could not be verified, because of the mutiple copies of these genes in the genome.

**Table 3:** Mutations discovered in evolved strains K1–K9

Genome position	Gene/IGR	Annotation/Product	Mutation	Variant ratio
Del514815A	CGB98_RS02465	16S ribosomal RNA	Del461A	8/9
T2052533C	CGB98_RS09630	HNH endonuclease	T291C	9/9
Del2720013A Del2720032T	CGB98_RS12800	IS6 family transposase	Del105T Del86A	7/9
In519737T	CGB98_RS02475-02480	5S ribosomal RNA copy B (*rrf*) and putative succinyl diaminopimelate transaminase (*dapC*), respectively	In-111T of *dapC*	8/9
A1791301G	CGB98_RS08395-08400	Peptidoglycan endopeptidase and cytochrome bc complex cytochrome b subunit, respectively	T-476C of RS08395	4/9
Del2206093A	CGB98_RS10415-10420	ABC transporter ATP-binding protein and 5S ribosomal RNA (*rrf*), respectively	Del-235T of RS10415	9/9
T2231742G	CGB98_RS10555-10560	tRNA-Lys and succinate CoA transferase (*actA*), respectively	A+20C of *actA*	4/9
Del2309538T	CGB98_RS10915-10920	Hypothetical protein and FAD-binding protein, respectively	Del+159A of RS10920	9/9

The other common mutations occurred in the intergenic region. These mutations may affect the expression of their downstream genes, i.e. *dapC*, CGB98_RS08395, and CGB98_RS10415, or their adjacent upstream genes, i.e. *actA* and CGB98_RS10920 (Table 3). Therefore, the transcription level of these genes in highest producer, i. e. K3 and K9 was verified by RT-PCR. Among these genes, only *dapC* are related to Lys biosynthetic pathway, while CGB98_RS08395 may promote cell wall degradation and affect the synthesis of peptidoglycan.

**Fig. 6:**
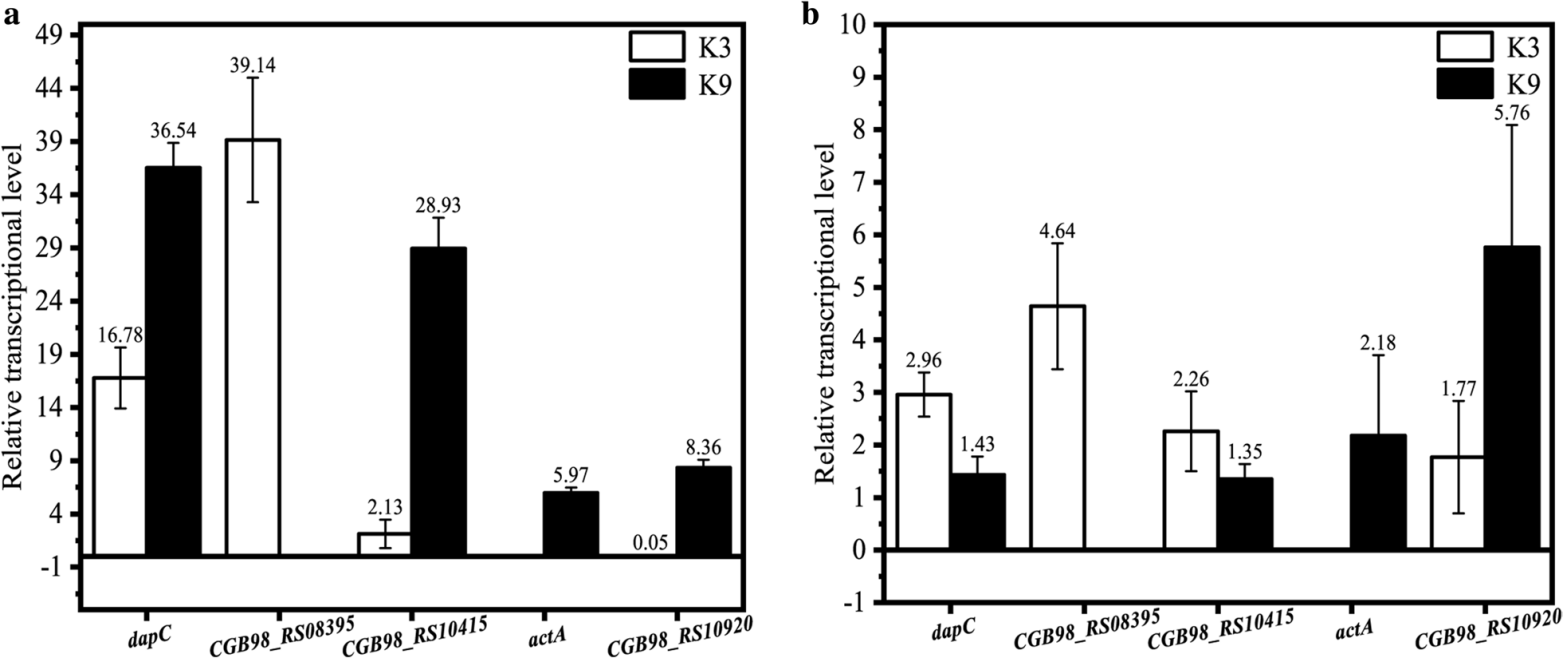
The relative transcription level of related genes in K3 and K9. **a** The relative transcription level at 24 h, **b** The relative transcription level at 48 h. The CGB98_RS08395 of K9 and *actA* of K3 were not mutated and thereby their transcription levels were not detected,

*dapC* encodes the *N*-succinyl-aminoketopimelate aminotransferase (DapC). It is dispensable for the synthesis of d, l-diaminopimelate via the succinylase branch. Other research demonstrated that overexpression of *dapC* resulted in a ninefold increase in this specific aminotransferase activity, along with an increase in Lys production (Hartmann et al. 2003). In K3 and K9, the relative transcription level of *dapC* increased significantly to 16.78- and 36.54-fold, respectively at 24 h as compared with SZ06 at 24 h (Fig. 6a), while after 48 h, their relative transcription level increased slightly to 2.96- and 1.43-fold of SZ06 (Fig. 6b). This might reslut in somewhat higher Lys accumulation in K3 and K9. In addition, the biomass of most mutant strains was a little higher than that of SZ06. The increased transcription level of the *dapC* gene might also affect the peptidoglycan synthesis and cell growth. CGB98_RS08395 encodes a peptidoglycan endopeptidases, proteins that are essential for promoting cell wall degradation and viability of bacterial cells (Shin et al. 2020). In K3, the transcription level of this gene significantly increased to 39.14-fold and 4.63-fold at 24 h and 48 h, respectively as compared with SZ06, in consistent with a higher biomass of K3.

CGB98_RS10415 encodes the ATP-binding protein of an ABC transporter. ABC transporters mainly mediate the absorption of essential nutrients into cells and the transport of molecules out of cells or into organelles, powered by ATP hydrolysis (Beis 2015). In K9, its transcription level at 24 h increased significantly to 28.93-fold as compared with SZ06, while in K3, its transcription level also increased to 2.13-fold. Both K3 and K9 strains performed a better growth, suggesting that this gene can promote the cell growth to some extents. *actA* encodes succinate CoA transferase which can supply succinyl CoA, an intermediate of TCA cycle and a precursor of Lys biosynthetic pathway. In K9, the transcription level of this gene significantly promoted to 5.97-fold and 2.18-fold as compared with SZ06 at 24 h and 48 h, respectively, thereby may increase the metabolic pool of TCA cycle and promote the running of TCA cycle, thus improving the 4-HIL production of mutant strains. Meanwhile, in the biosynthetic pathway of Lys, DapC converts a succinylated intermediate and succinyl CoA is required as the substrate by DapD for synthesizing this succinylated intermediate. The promoted expression of *actA* and supply of succinyl CoA may also enhance the Lys biosynthesis, thereby increasing the Lys accumulation in K9. CGB98_RS10920 encodes a FAD-binding protein. In K9, the relative transcription level of this gene rised to 8.36-fold and 5.76-fold of that SZ06 at 24 and 48 h, respectively. While in K3, its transcriptional level decreased greatly at 24 h and increased significantly at 48 h. In conclusion, this programming ALE generated a total of 290 mutations in 9 evolved strains, and most of these mutations are independent of the 4-HIL biosynthetic pathway, thereby may be the non-intuitive mutations. Only *dapC* is related directly to Lys biosynthetic pathway, while *actA* is involved in the synthesis of succinyl CoA and thereby influences Lys biosynthesis. The upregulation of *dapC* and *actA* in these evolved strains would result in the accumulation of Lys. The increased expression of CGB98_RS08395 may affect cell survival and the sysnthesis of peptidoglycan.

## Discussion

ALE is a powerful strategy in metabolic engineering to significantly improve growth performance and phenotypes of strains. Some studies have utilized ALE to force strains to adapt to alternative growth environments and to obtain mutants with significantly improved performance. For example, a *C. glutamicum* strain with improved tolerance to high concentrations of methanol was successfully obtained by ALE, thereby enhancing methanol biotransformation, meanwhile, the cell growth was also improved (Tuyishime et al. 2018; Wang et al. 2020). ALE was successfully applied for improving the tolerance of *E. coli* to sabinene, and the sabinene production was increased to 8.43-fold (Wu et al. 2020). ALE was also applied for generating a *C. glutamicum* strain with higher growth rates on glucose minimal medium (Pfeifer et al. 2017). But the mutation rate is too low when ALE is performed under natural evolution, thereby much long time is required for running such ALE cycle. It usually takes several months, such as in the case that *C. glutamicum* ATCC 13032 was adapted on glucose minimum medium for more than 1500 generations, i.e. approximately 7 months, and finally several faster growing mutants were isolated (Wang et al. 2018). The shortest time for ALE is about two weeks, for example, *C. glutamicum* strains with improved utilization of D-xylose were obtained by using miniaturized and automated ALE within 13 days (Radek et al. 2017). Furthermore, if the selection pressure coupled to growth is not available, the desired mutant strains are difficult to be screened out by ALE. Thus, high-throughput screening, such as FACS, is usually designed for a specific phenotype, because the target metabolites can be visualized and the high-production strains can be obtained after extensive screening. The LysG-P_*lysE*_ or Lrp-P_*brnFE*_ biosensor-driven ALE followed by FACS had been used to isolate new high-producing *C. glutamicum* mutants (Binder et al. 2012; Mahr et al. 2015). However, FACS requires a large number of screenings to obtain the desired phenotype, and the application of FACS is limited by equipment and instrument.

In order to improve the mutation rate and reduce the screening effort, here, a Lys-sensing evolution system was constructed to accelerate and programme ALE process, and to further realize the controllability of the mutation rate. In the actuator, the mutagenesis gene *cdd* under the control of LysG-P_*lysE*_ biosensor was used to accelerate mutation and evolution, and the eGFP fluorescence intensity of cells was detected to monitor the evolutionary progress according to the intracellular Lys concentration. After eight rounds of evolution, the positive mutation rate based on 4-HIL titer reached 95.5%, while after three rounds of evolution, it reached 54.5%. Meanwhile, the later positive mutants produced a little more 4-HIL (54.93 − 79.67 mM) as compared with the former positive mutants (43.31 − 63.98 mM). The biomass and glucose consumption of all the nine best mutant strains K1 − K9 were nearly same as SZ06, among them, the growth performance of K3 and K9 was slightly better. K1 − K9 accumulated 128.87–152.19 mM 4-HIL in shake flask, all higher than SZ06 (118.56 ± 7.21 mM). Among them, K3 reached the highest 4-HIL titer of 152.19 ± 14.60 mM and this is the highest titer reported so far. Compared to eight rounds of evolution, three rounds can also achieve mutant strains with higher 4-HIL production in a shorter evolution time. Regrettably, the Lys accumulation did not significantly reduce by this programming ALE, although the relative eGFP fluorescence intensity at the eighth round of evolution decreased. Such inconsistency may be partially due to the increased biomass after eight rounds of evolution (Fig. 1d). The difficulty for reducing Lys content is probably because that the Lys biosynthetic pathway is important for cell growth and peptidoglycan synthesis (Wehrmann et al. 1998; Hutton et al. 2007; Hochheim et al. 2017). The increased expression of *dapC* and *actA* in our final evolved strains also verified the essential of Lys biosynthesis. In addition, some non-intuitive mutations generated by this programming ALE occurred in ribosomal genes, such as 16S or 5S ribosomal RNA genes. The mutations in these genes might accelerate the overall translation process of cells and thus improve their metabolic ability to synthesize 4-HIL and Lys.

In addition, programming ALE can cause numerous mutations as compared to natural evolution. Natural evolution produces nondirectional mutations that require more effort to screen and identify relevant targets, but there are only a few meaningful mutations. Recently, a metabolic engineering to guide evolution (MGE) was applied to improve Val producing strains by creating the evolutionary pressure and to get more beneficial mutations (Schwentner et al. 2018). In our study here, the programming ALE generated a total of 290 mutations in 9 evolved strains, and these mutations were almost unrelated to the 4-HIL biosynthetic pathway. However, a mutant strain with significantly increased 4-HIL titer, i.e. K3 was finally obtained. These results verify the idea that programming ALE driven by the Lys-sensing evolutionary system can increase the mutation rate, shorten the evolution time, and generate mutant strains with better phenotype. Although the LysG-P_*lysE*_ biosensor cannot be accurately characterized in our programming ALE system, it can be confirmed at least that the LysG-P_*lysE*_ biosensor can modulate the expression of its downstream gene(s) to a certain extent under different Lys levels, as proven by a research that Lys acted as an inducer to mediate the transcriptional activation ability of LysG, thereby controlling the expression of the downstream gene *lysE* (Bellmann et al. 2001).

A research that improved 4-HIL titer by seven steps of static metabolic engineering combined with one step of dynamic control finally generated a recombinant *C. glutamicum* strain YI (Zhang et al. 2018). Their modification contained codon optimization, gene deletion, promoter substitution, gene overexpression, dynamic modulation of emzyme activity, and so on, and it took long time to finish all modification and to obtain the final recombinant strain. In contrast, the Lys-sensing evolution system pMK can be constructed quickly within only 2 days, while the evolved strain K1 − K9 can be obtained within 20 − 30 days. This design successfully achieved the highest 4-HIL producer at the shake flask level. The use of the programming ALE driven by Lys-biosensor opens new perspectives for the development of *C. glutamicum* strains with higher 4-HIL production. The programming ALE will be more powerful than the traditional evolution or metabolic engineering in the future. Regrettably, the concentration of by-product Lys did not reduce. The Lys biosensor LysG-P_*lysE*_ will be optimized so that its sensitivity toward Lys can better adapt the 4-HIL fermentation environment and drive the programming ALE to reduce the Lys content. Meanwhile, other strain with less Lys accumulation can be selected as the initial evolutionary strain.

## Data Availability

All data are included in the manuscript.

## Abbreviations

ALE: Adaptive laboratory evolution
Arg: L-arginine
BACCs: Branched-chain amino acids
Ct: Cycle threshold
FACS: Fluorescence-activated cell sorting
FREP: Feed-back regulated evolution of phenotype
4-HIL: 4-Hydroxyisoleucine
His: L-histidine
IDO: L-isoleucine dioxygenase
Ile: L-isoleucine
InDel: Insertion-deletion
α-KG: α-Ketoglutarate
LB: Luria–Bertani
Lys: L-lysine
RT-PCR: Real-time PCR
SNP: Single nucleotide polymorphisms
T2DM: Type 2 diabetes mellitus
TF: Transcription factor
Val: L-valine
